# Expanded *CRB2*-related disease phenotype: multisystem involvement and post-transplant complications in monozygotic twins

**DOI:** 10.1007/s00467-025-06827-w

**Published:** 2025-06-03

**Authors:** Moran Plonsky Toder, Shirley Pollack, Rami Tibi, Irina Libinson-Zebegret, Renata Yakubov, Israel Eisenstein, Mika Shapira Rootman, Daniella Magen

**Affiliations:** 1 Technion Israel Institute of Technology, Rappaport Faculty of Medicine, Haifa, Israel; 2 Ruth Rappaport Children’s Hospital, Rambam Health Care Campus, Pediatric Nephrology Institute, Haifa, Israel

**Keywords:** Congenital nephrotic syndrome, *CRB2* mutation, Pediatric kidney transplantation

## Abstract

**Background:**

Congenital nephrotic syndrome (CNS) is a rare disorder caused by mutations in genes essential for podocyte function and glomerular slit diaphragm integrity, including *CRB2* (Crumbs Cell Polarity Complex Component 2). *CRB2* mutations are linked to focal segmental glomerulosclerosis and ventriculomegaly with cystic kidney disease, but their full phenotypic spectrum remains unclear. We describe the clinical course of monozygotic twins with a homozygous *CRB2* mutation, highlighting severe complications following kidney transplantation.

**Methods:**

The twins, who were followed and managed throughout their clinical course, were diagnosed with CNS after prenatal suspicion of polycystic kidney disease. Initial exome sequencing was negative, but subsequent whole exome sequencing revealed a homozygous *CRB2* variant.

**Results:**

Both twins presented with CNS, requiring intensive supportive care. Additional findings included cerebral heterotopia, cardiac involvement, and developmental delay. They both progressed to kidney failure, necessitating hemodialysis in early childhood. Post-transplant, the first twin succumbed to a systemic fungal infection, while the second developed complications linked to immune dysregulation, including post-transplant lymphoproliferative disease (PTLD), immune thrombocytopenic purpura (ITP), multiple viremias, and de novo donor-specific antibodies (DSA).

**Conclusions:**

This case expands the phenotypic spectrum of *CRB2*-related disease, highlights management challenges, and underscores the need for genetic re-analysis in rare diseases. Further research is required to understand *CRB2*-related mechanisms.

**Graphical abstract:**

**Figure:**
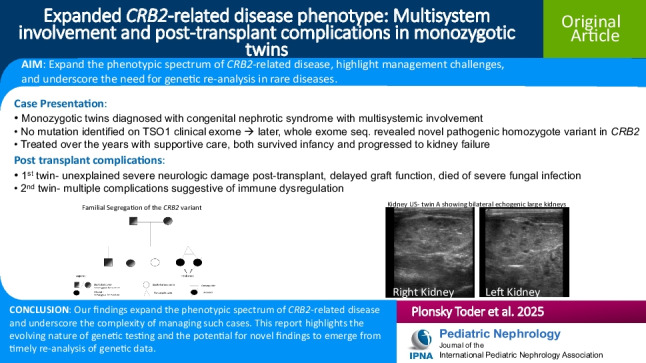
A higher resolution version of the Graphical abstract is available as Supplementary information

**Supplementary Information:**

The online version contains supplementary material available at 10.1007/s00467-025-06827-w.

## Introduction

Congenital nephrotic syndrome (CNS) is a rare condition that may result from genetic mutations disrupting podocyte function and the glomerular slit diaphragm, resulting in severe early-onset proteinuria, hypoalbuminemia, and edema [1]. The most implicated genes in CNS include *NPHS1*, *NPHS2*, *WT1*, and *PLCE1*. Advances in sequencing technologies continue to uncover additional, rarer causes of CNS, broadening our understanding of its genetic landscape [1–3].

Among these less frequent genes associated with CNS, *CRB2* (Crumbs Cell Polarity Complex Component 2) encodes a protein family involved in critical cellular processes during early embryonic development [4–6]. Mutations in *CRB2* have been linked to focal segmental glomerulosclerosis type 9 (FSGS9) and ventriculomegaly with cystic kidney disease [6, 7].

The first reports of *CRB2*-associated steroid-resistant nephrotic syndrome (SRNS) emerged in 2015, when Slavotinek et al. described six patients from three families exhibiting nephrosis, ventriculomegaly, and elevated alpha-fetoprotein levels, with prenatal manifestations being a hallmark. Five pregnancies were terminated due to extensive hydrocephalus, while the sixth infant succumbed at 7 months of age to uncontrolled nephrotic syndrome.

Simultaneously, Ebarasi et al. reported four families with *CRB2*-related SRNS [7] presenting between 9 months and 6 years of age. While kidney biopsies revealed FSGS histology, their clinical course was not described in detail. Lamont et al. [8] expanded the phenotypic spectrum of the disease, reviewing earlier cases and adding five more individuals with predicted pathogenic biallelic *CRB2* variants. Common findings included ventriculomegaly and kidney involvement. However, specific clinical manifestations and kidney outcomes remained largely unexplored. Other features included mild ocular abnormalities and one case of B cell lymphoma.

Subsequent case reports described several children with *CRB2* mutations who developed SRNS without progression to kidney failure [9, 10, 10, 11], highlighting the heterogeneity of *CRB2*-related phenotypes. The largest cohort to date, by Aduteum et al. [12], further refined the disease manifestations. This study identified kidney involvement, primarily nephrotic syndrome (NS) without kidney failure or early demise, as the predominant feature, often accompanied by central nervous system and cardiac anomalies. Most pathogenic variants were located in exons 8 and 10 of the *CRB2* gene, while variants in exons 12 and 13 were more commonly associated with isolated kidney manifestation [12]. Notably, only one patient in this cohort progressed to advanced chronic kidney disease (CKD), reaching CKD stage 5 necessitating kidney transplantation at 10 years of age.

Despite these findings, there is still a considerable gap in understanding the clinical trajectory of *CRB2*-associated CNS beyond infancy, especially regarding progression to kidney failure or outcomes following kidney transplantation.

Here, we describe two monozygotic diamniotic twin girls with a novel homozygous pathogenic *CRB2* mutation. These twins exhibited a complex phenotype, survived into childhood, progressed to kidney failure, and encountered severe and uncommon post-transplant complications. This report provides novel insights into the long-term clinical trajectory of *CRB2*-related disorders.

## Methods: patient descriptions

### Initial clinical course

Our patients are monozygotic twin girls, born to consanguineous parents, with no known family history of kidney disease. During pregnancy, elevated alpha-fetoprotein levels (MoM 63) raised concerns about potential fetal anomalies. Whole body fetal ultrasound was largely normal for both twins, except for mildly enlarged, hyperechogenic kidneys and mild oligohydramnios in Twin A, raising suspicion for polycystic kidney disease.

The twins were delivered prematurely at 36 weeks gestation, following spontaneous labor. Both were small for gestational age, with birth weights below the 10 th percentile (1870 g and 2185 g, respectively). Despite prematurity, they required minimal respiratory support and were subsequently stabilized in the neonatal intensive care unit.

#### Twin A

**Fig. 1:**
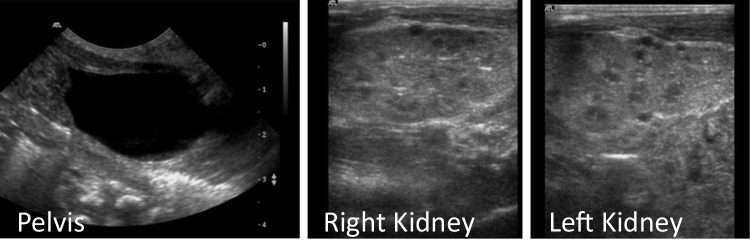
Kidney ultrasound of Twin A during the neonatal period showing bilateral kidney enlargement, increased echogenicity, and multiple cysts

Postnatal ultrasounds confirmed the prenatal findings of bilateral kidney enlargement, hyperechogenicity, and multiple cysts, further supporting the suspected diagnosis of polycystic kidney disease (Fig. 1). She also had mild hyperkalemia without metabolic acidosis and mildly elevated serum creatinine levels, managed conservatively. Early in infancy, she developed nephrotic syndrome, requiring regular albumin infusions via a central line catheter, as well as additional supportive care including thyroxine supplementation, preventive anticoagulation, ACE inhibitors, and diuretics.

Other notable phenotypic features included dilated cardiomyopathy with mitral regurgitation, ventriculomegaly, and neuronal migration abnormalities, presenting as cerebral gray matter heterotopy on brain imaging. Ocular examination excluded signs of retinitis pigmentosa. At 2.8 years of age, she progressed to kidney failure, and hemodialysis was initiated. Although her cardiomyopathy was well controlled with antihypertensive medication, she experienced exacerbations during acute illnesses. These episodes manifested as a decline in cardiac function, necessitating temporary inotropic support and treatment with levosimendan (a calcium sensitizer used in the management of acutely decompensated congestive heart failure). She also exhibited global developmental delay. Otherwise, she remained stable on a standard hemodialysis regimen. At 4.5 years of age, she underwent a deceased donor kidney transplantation.

#### Twin B

**Fig. 2:**
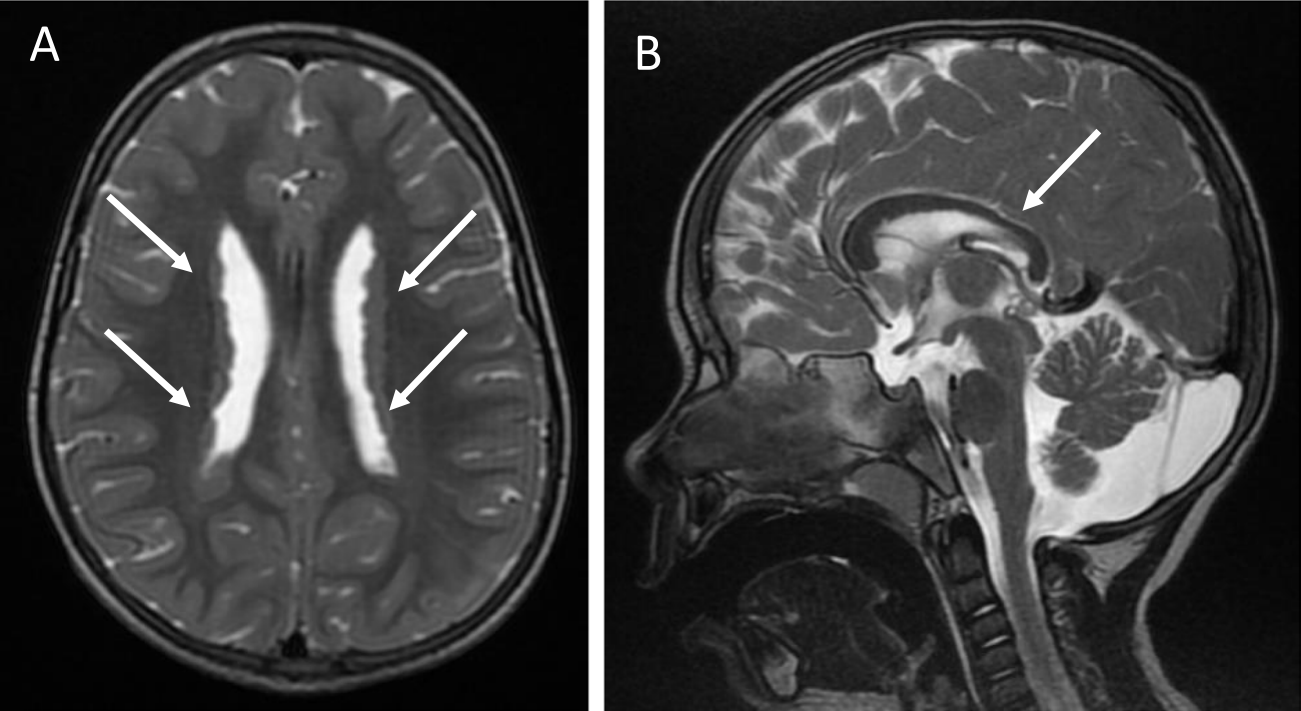
Brain imaging findings from MRI performed at age 4 (Twin B). **A** Axial T2-weighted images: Bilateral symmetric subependymal heterotopia is noted (arrows). The ventricles are normal in size, with no signs of hydrocephalus. **B** Midline sagittal T2-weighted image: The corpus callosum is slightly short, with mild thinning observed in the posterior portion, including the posterior body, isthmus, and splenium (arrow). In the posterior fossa, the vermis is relatively small (cranio-caudal dimensions), displaying features consistent with inferior vermian hypoplasia. Additionally, prominent CSF spaces and an enlarged cisterna magna are present in the posterior fossa

Although prenatal ultrasound was apparently unremarkable, postnatal assessment revealed mildly echogenic kidneys without cysts. Like her sister, she developed nephrotic syndrome in infancy, requiring regular albumin infusions as well as supportive care. She exhibited milder manifestations of cardiomyopathy, ventriculomegaly, and neuronal migration issues (Fig. 2), as well as milder developmental delay, compared to Twin A. She also had no signs of retinitis pigmentosa. At 3.5 years, she developed kidney failure and hemodialysis was initiated. During dialysis sessions, she frequently experienced headaches though no other significant issues were noted. At 5.5 years, she underwent a deceased donor kidney transplantation.

### Genetic analysis

As part of our Nephrogenetic Initiative, genetic testing is routinely offered to our patients with kidney failure suspected to suffer from a monogenic disorder, particularly in the presence of consanguinity, familial nephropathy, or a clinical presentation indicative of a specific genetic disorder. Following the twins’ presentation, initial testing using the TruSight One clinical exome sequencing panel (Illumina, San Diego, CA) failed to reveal any suspected disease-causing variants. However, given their unique phenotype and the known consanguinity, we pursued a broader genetic re-analysis, using whole exome sequencing (WES), which was performed when it became available to us, 2 years later. Sequencing was conducted on a Novaseq6500 platform using the IDT_xGen_Exome_Research_Panel_v2 kit (IDT, Coralville, Iowa). The resulting reads were mapped to the reference genome (build GRCh37/hg19), and variant calling, annotation, and data analysis were carried out using the Genoox data analysis platform (Genoox, Palo Alto, CA).

WES analysis identified a homozygous missense variant in exon 7 of the *CRB2* gene: c.1813 C > T; p.Arg605 Cys (Chr9: g126133145 C > T; NM_173689.6). Located within the EGF-like domain, this variant replaces a semi-conserved arginine (Arg) with cysteine (Cys), which is predicted to alter the physical properties of the encoded product. Arg-to-Cys substitutions in EGF-like domains have previously been reported as pathogenic [7]. This specific alteration has been observed in a heterozygous state in four individuals in the gnomAD database (https://gnomad.broadinstitute.org/), but has not been reported in ClinVar (https://www.clinicalgenome.org/data-sharing/clinvar/). Although prediction tools provided conflicting assessments regarding its suspected pathogenicity, the p.Arg605 Cys was classified as “likely pathogenic” according to ACMG 2015 and ClinGen guidelines. Of note, since the *CRB2* gene was not included in the TruSight sequencing panel, it was omitted from our initial genetic analysis.

**Fig. 3:**
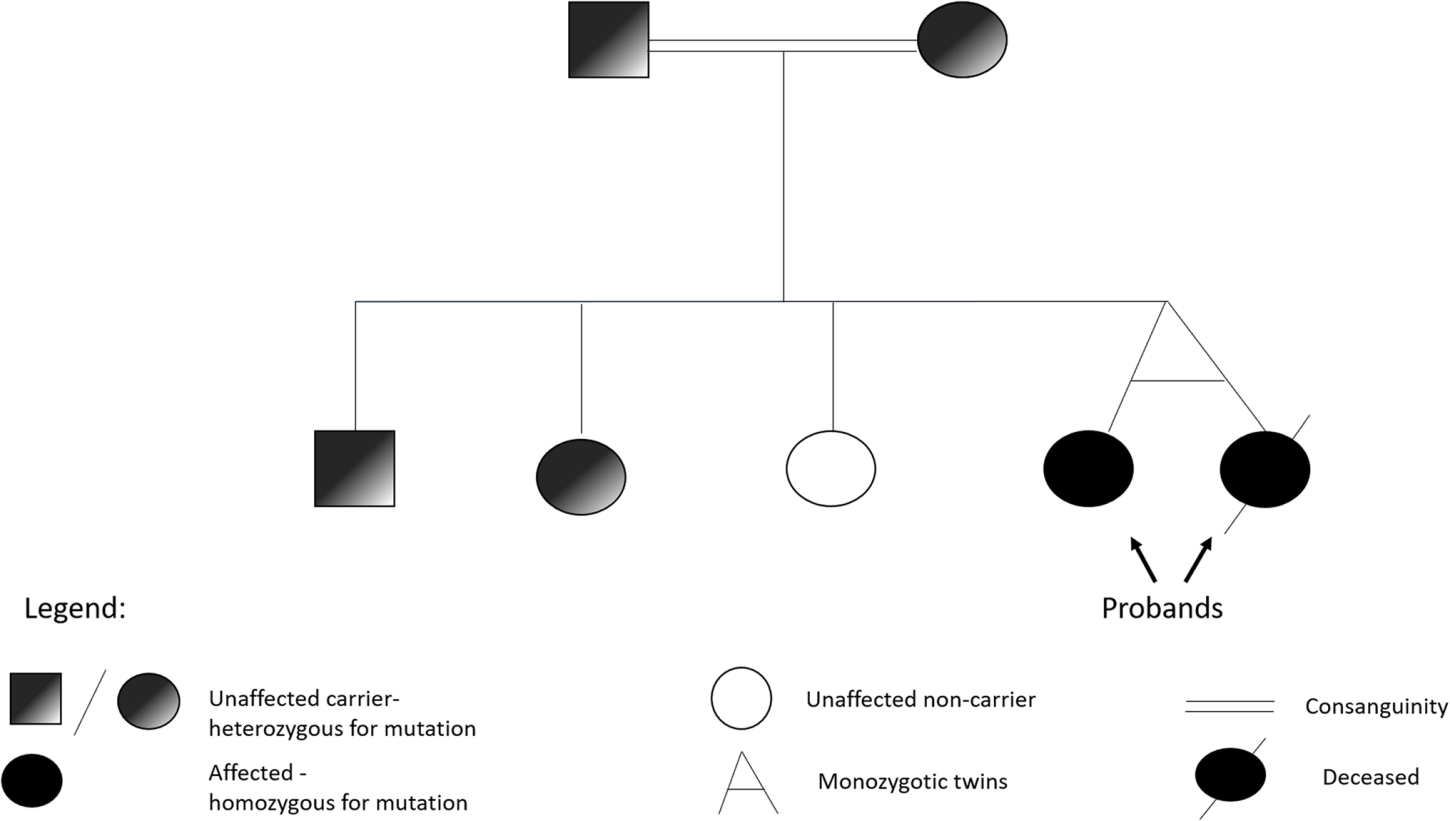
Familial segregation of the *CRB2* variant

The p.Arg605 Cys variant was confirmed in both twins by Sanger sequencing. Family segregation analysis confirmed an autosomal recessive mode of inheritance, as both healthy parents and two of the three healthy siblings were asymptomatic heterozygous carriers, while one sibling showed a wild-type sequence (Fig. 3).

Combining the distinct clinical phenotype of the twins, which aligns with previously reported cases of *CRB2-*related disease, the rarity of this variant, and the confirmed family segregation, we proposed that p.Arg605 Cys is a novel disease-causing variant in the *CRB2* gene.

### Subsequent medical course

#### Twin A

At the age of 3.5 years, she underwent a deceased donor kidney transplant, prior to the identification of the underlying genetic etiology. The induction regimen included basiliximab and methylprednisolone, while maintenance therapy was planned with prednisone, MMF, and tacrolimus, according to standard protocols. Although the surgical procedure was uneventful, she developed an unexplained neurological deficit post-transplantation, failing to regain full post-anesthesia consciousness. Head CT revealed diffused cerebral edema of unknown etiology. In addition, the patient experienced delayed graft function, requiring short-term dialysis.

Given the suspected role of calcineurin inhibitor (CNI) toxicity in her neurological complications, despite an average tacrolimus level of 7.25 ng/mL (ranging from 3.3 to 19 ng/mL), which is not considered extremely high, tacrolimus was discontinued. However, due to her delayed graft function and the growing concern for acute rejection following CNI cessation, and despite negative donor-specific antibodies (DSA) and the absence of biopsy evidence of rejection, antithyroglobulin therapy (7.5 mg/kg) was administered.

Her maintenance regimen was adjusted to include prednisone, MMF, and everolimus (mTOR inhibitor). While kidney function subsequently improved, she developed surgical wound dehiscence followed by an invasive fungal infection. Unfortunately, she did not respond to broad-spectrum antifungal therapy and ultimately succumbed to a disseminated fungal infection 3 months post-transplant, following a prolonged and challenging intensive care hospitalization. No external trigger was identified to explain her neurological damage, raising the possibility of rare CNI-related toxicity.

#### Twin B

Nearly 2 years after the demise of her twin, she received a deceased donor kidney transplant. To reduce the risk of neurological complications potentially associated with tacrolimus, she was initially managed with a tacrolimus-free immunosuppressive protocol. Induction included antithyroglobulin and methylprednisolone, with maintenance therapy using prednisone, MMF, and everolimus. Her peri-transplant course was uneventful, aside from a mild subdural hematoma and CSF leakage from the epidural analgesia, which was successfully managed with a therapeutic blood patch. Mild proteinuria led to an eventual switch from everolimus to tacrolimus, without additional complications. Over the following years, she developed multiple uncommon complications indicative of immune dysregulation, presenting both under and over immunosuppression, as follows:

- De novo DSA (Class II): High mean fluorescence intensity (MFI) levels (9000) appeared 2 months post-transplant. She was managed with everolimus, switched to tacrolimus, and with IVIG as well as rituximab, but DSA levels persisted.
- CMV viremia: Suspected gastrointestinal CMV disease developed 5 months post-transplant. It was refractory to IV ganciclovir, but responded to foscarnet.
- EBV-related post-transplant lymphoproliferative disorder (PTLD): EBV-associated PTLD was diagnosed 6 months post-transplant. EBV PCR in the blood became positive only after the diagnosis of PTLD, with tumor tissue biopsy confirming EBV involvement and excluding CMV. MMF was discontinued, and she responded well to rituximab therapy.
- Celiac disease: Diagnosed 6 months post-transplant and managed with a gluten-free diet.
- Pseudotumor cerebri: Diagnosed 2 years post-transplant and treated with Diamox, which resulted in severe tubulopathy necessitating the discontinuation of the medication, with no recurrence.
- Immune thrombocytopenic purpura (ITP): Gastrointestinal bleeding occurred 2 years post-transplant. Steroids and IVIG were ineffective, but she responded well to four doses of rituximab.
- Chronic leukopenia: Persistent mild-to-moderate leukopenia with intermittent neutropenia and lymphopenia was observed throughout the entire post-transplant period.
- Multiple infections: Since transplantation, she has experienced viral and bacterial infections, including frequent urinary tract infections, which remain an ongoing issue.

Currently, 3 years post-transplant, she remains stable with normal graft function on a dual immunosuppressive regimen of prednisone and tacrolimus. The reintroduction of MMF was avoided due to opportunistic viral infections and her history of PTLD. Following multiple doses of IVIG, her DSA levels have finally declined without intensifying immunosuppression.

## Discussion

We present monozygotic girls born to consanguineous parents, both harboring a potentially harmful homozygous missense variant in *CRB2*. Their phenotype aligns with the known features of *CRB2*-related features, including cystic kidney disease, early-onset nephrosis, ventriculomegaly, gray matter heterotopia, and cardiomyopathy [6–8, 12, 13]. Notably, retinitis pigmentosa, reported in other cases, was absent.

When considering the genotype–phenotype correlation in *CRB2*-associated conditions, the affected domain does not fully explain the phenotype. However, specific mutations typically result in either solitary SRNS or a broader *CRB2*-related disease, but not both [14]. Notably, at least one variant (*p.Gly1036 Alafs*43*) has been reported in both *CRB2*-related disease (four individuals) and in SRNS (one individual), challenging the notion of mutually exclusive variants [12]. Moreover, no clear correlation exists between the severity of kidney disease and the extent of systemic involvement [14]. It has been suggested that the most pathogenic variants causing *CRB2*-related disease are overrepresented in exons 8 and 10 of the *CRB2* gene, while variants in exons 12 and 13 are more commonly linked to isolated kidney manifestations [12]. Regarding exon 7, implicated in our case, previously reported mutations in this exon have been associated with either isolated kidney involvement or a broader multisystemic phenotype, with no phenotypic overlap among identical mutations. Mutations in this exon typically affect either the EGF-like domain, as in our case, or the Laminin G domain [12]. While the only reported mutation in the Laminin G domain was linked to *CRB2*-related syndrome, mutations in the EGF-like domain have been associated with both phenotypes [12]. Here, we report a novel homozygous pathogenic variant in the EGF-like domain of exon 7 of the *CRB2* gene, associated with a broad multisystemic phenotype, including nephrosis, CNS abnormalities, and cardiac involvement. This newly identified homozygous variant expands the existing database and may ultimately contribute to a better understanding of the genotype–phenotype correlation in this rare condition.

Interestingly, despite their identical genetic makeup and shared in utero environment, the twins exhibited significant phenotypic heterogeneity in symptom onset, severity, and post-transplant outcome. This variability suggests the influence of additional factors, both genetic and non-genetic, including epigenetic changes, the broad phenotypic spectrum of *CRB2*-related disease, and interactions with environmental, infectious, and drug-related factors.

Unlike most previously reported cases, which often resulted in pregnancy termination or early neonatal demise due to severe hydrocephalus or seizures, both twins survived infancy. This may be attributed to the absence of severe CNS manifestations, such as severe hydrocephalus or intractable seizures (as described in previous reports), manageable nephrotic syndrome, and their relatively stabilized cardiomyopathy.

Both girls survived the neonatal and infantile periods, progressed to kidney failure at a young age (2.8 and 3.5 years), and were treated with hemodialysis followed by deceased donor kidney transplantation.

Twin A experienced a catastrophic post-transplant course, marked by unexplained neurological complications, likely multifactorial, and a severe fungal infection. Despite extensive evaluation, no clear trigger or explanation was identified. Her head CT and subsequent MRI revealed known gray matter heterotopia and suspected anoxic changes, though no obvious hypoxic event was observed. Potential causes included CNI toxicity, an unrecognized vascular event, or status epilepticus, but none was supported by solid evidence. Her unfortunate demise due to the fungal infection can be attributed to immunosuppression, though an underlying predisposition may have exacerbated her condition.

Twin B had a relatively stable peri-transplant course but developed a cascade of immunological and infectious complications over time, including high DSA levels, PTLD, CMV disease, and ITP.

While previous descriptions of patients with *CRB2* disruption lack reports of infectious or immunologic complications or data on transplant outcomes, a documented case of B cell lymphoma in a young child—resulting in death at 3 years of age due to chemotherapy complications [8]—raises suspicion of a potential link between the underlying genetic defect and immune dysregulation.

This exceptionally atypical clinical course in our experience further supports the likelihood of an underlying cause connected to their known genetic mutation.

The *CRB2* gene, a member of the Crumbs family of apical polarity-related transmembrane proteins, plays a critical role in maintaining cellular polarity, adhesion, mechanotransduction, and intracellular signaling in epithelial cells, particularly podocytes and neuronal cells. Emerging evidence suggests that *CRB2* disruption may contribute to immune dysregulation through mechanisms involving cellular stress responses, apoptosis, and signaling pathways. Specifically, *CRB2* deficiency leads to increased YAP activity, dysregulated sphingosine 1-phosphate receptor (S1PR1) expression, and enhanced apoptotic signaling [15]. While RNA sequencing and microarray analyses have reported *CRB2* expression in immune-related tissues including whole blood, thymus, lymph nodes, and EBV-driven lymphocytes (GeneCards.org, Franklin by Genoox), no studies have directly explored its role in immune cell function. Further research is needed to determine whether *CRB2* directly regulates immune cells or indirectly influences immune responses through its effects on epithelial integrity and mechanosensing.

This case expands the phenotypic spectrum of *CRB2*-associated disease, demonstrating survival beyond infancy, progression to kidney failure, and highly complex transplant outcomes. The additional features of cardiomyopathy, ventriculomegaly, and neuronal migration defects further illustrate the broad spectrum of manifestations linked to *CRB2* mutations. This finding underscores the importance of broader genetic testing in the diagnosis and management of congenital kidney diseases. As new genetic discoveries emerge and sequencing technologies evolve, repeated or expanded testing may be necessary to identify previously undetected disease-causing variants, as demonstrated in this case, where the *CRB2* mutation was initially missed by TruSight One clinical exome sequencing (Illumina, San Diego, CA) but later identified through whole exome sequencing. Moreover, the contrasting post-transplant courses in these twins emphasize the need for individualized, cautious management and underscore the urgency for improved immunological markers to guide anti-rejection regimens and to enable individualized immunosuppressive therapy. Further studies on the natural history and transplant outcomes in patients with *CRB2* mutations will be essential to advance our understanding of this rare disorder.

## Supplementary Information

Below is the link to the electronic supplementary material.

Graphical abstract (PPTX 1.07 MB)

## Data Availability

All data generated or analyzed during this study are included in this published article.

## References

[1] Boyer O, Schaefer F, Haffner D, Bockenhauer D, Hölttä T, Bérody S, Webb H, Heselden M, Lipska-Zie˛tkiewicz BS, Ozaltin F, Levtchenko E, Vivarelli M (2021) Management of congenital nephrotic syndrome: consensus recommendations of the ERKNet-ESPN Working Group. Nat Rev Nephrol 17:277–289. 10.1038/s41581-020-00384-1 10.1038/s41581-020-00384-1 PMC8128706 33514942

[2] AbuMaziad AS, Abusaleh R, Bhati S (2021) Congenital nephrotic syndrome. J Perinatol 41:2704–2712. 10.1038/s41372-021-01279-0 34983935 10.1038/s41372-021-01279-0

[3] Sharma J, Saha A, Ohri A, More V, Shah F, Dave J, Jain BP, Matnani M, Sathe K, Bhansali P, Chhajed P, Deore P, Pande N, Shah C, Kinnari V, Singhal J, Krishnamurthy N, Agarwal M, Ali U (2024) New insights from the genetic work-up in early onset nephrotic syndrome: report from a registry in western India. Pediatr Nephrol 39:2099–2104. 10.1007/s00467-024-06295-8 38294522 10.1007/s00467-024-06295-8

[4] Hamano S, Nishibori Y, Hada I, Mikami N, Ito-Nitta N, Fukuhara D, Kudo A, Xiao Z, Nukui M, Patrakka J, Tryggvason K, Yan K (2018) Association of crumbs homolog-2 with mTORC1 in developing podocyte. PLoS One 13:e0202400. 10.1371/journal.pone.0202400 30125302 10.1371/journal.pone.0202400 PMC6101391

[5] Möller-Kerutt A, Rodriguez-Gatica JE, Wacker K, Bhatia R, Siebrasse J-P, Boon N, Van Marck V, Boor P, Kubitscheck U, Wijnholds J, Pavenstädt H, Weide T (2021) Crumbs2 is an essential slit diaphragm protein of the renal filtration barrier. J Am Soc Nephrol 32:1053–1070. 10.1681/ASN.2020040501 33687977 10.1681/ASN.2020040501 PMC8259666

[6] Slavotinek A, Kaylor J, Pierce H, Cahr M, DeWard SJ, Schneidman-Duhovny D, Alsadah A, Salem F, Schmajuk G, Mehta L (2015) CRB2 mutations produce a phenotype resembling congenital nephrosis, Finnish type, with cerebral ventriculomegaly and raised alpha-fetoprotein. Am J Hum Genet 96:162–169. 10.1016/j.ajhg.2014.11.013 25557780 10.1016/j.ajhg.2014.11.013 PMC4289687

[7] Ebarasi L, Ashraf S, Bierzynska A, Gee HY, McCarthy HJ, Lovric S, Sadowski CE, Pabst W, Vega-Warner V, Fang H, Koziell A, Simpson MA, Dursun I, Serdaroglu E, Levy S, Saleem MA, Hildebrandt F, Majumdar A (2015) Defects of CRB2 cause steroid-resistant nephrotic syndrome. Am J Hum Genet 96:153–161. 10.1016/j.ajhg.2014.11.014 25557779 10.1016/j.ajhg.2014.11.014 PMC4289689

[8] Lamont RE, Tan WH, Innes AM, Parboosingh JS, Di S-D, Rajkovic A, Pappas J, Altschwager P, Deward S, Fulton A, Gray KJ, Krall M, Mehta L, Rodan LH, Saller DN, Steele D, Stein D, Yatsenko SA, Bernier FP, Slavotinek AM (2016) Expansion of phenotype and genotypic data in CRB2-related syndrome. Eur J Hum Genet 24:1436–1444. 10.1038/ejhg.2016.24 27004616 10.1038/ejhg.2016.24 PMC5027675

[9] Lu J, Guo Y-N, Dong L-Q (2021) Crumbs homolog 2 mutation in two siblings with steroid-resistant nephrotic syndrome: two case reports. World J Clin Cases 9:3056–3062. 10.12998/wjcc.v9.i13.3056 33969091 10.12998/wjcc.v9.i13.3056 PMC8080757

[10] Simaab A, Krishin J, Alaradi SR, Haider N, Shah M, Ullah A, Abdullah A, Ahmad W, Hansen T, Basit S (2022) Exome sequencing revealed a novel splice site variant in the CRB2 gene underlying nephrotic syndrome. Med Kaunas Lith 58:1784. 10.3390/medicina58121784 10.3390/medicina58121784 PMC9781877 36556986

[11] Yang Q, Tang D, Gan C, Bai M, Song X, Jiang W, Li Q, Chen Y, Zhang A, Wang M (2024) Novel variants in CRB2 targeting the malfunction of slit diaphragm related to focal segmental glomerulosclerosis. Pediatr Nephrol 39:149–165. 10.1007/s00467-023-06087-6 37452832 10.1007/s00467-023-06087-6

[12] Adutwum M, Hurst A, Mirzaa G, Kushner JD, Rogers C, Khalek N, Cristancho AG, Burrill N, Seifert ME, Scarano MI, Schnur RE, Slavotinek A (2023) Six new cases of CRB2-related syndrome and a review of clinical findings in 28 reported patients. Clin Genet 103:97–102. 10.1111/cge.14222 36071576 10.1111/cge.14222

[13] Jaron R, Rosenfeld N, Zahdeh F, Carmi S, Beni-Adani L, Doviner V, Picard E, Segel R, Zeligson S, Carmel L, Renbaum P, Levy-Lahad E (2016) Expanding the phenotype of CRB2 mutations - a new ciliopathy syndrome? Clin Genet 90:540–544. 10.1111/cge.12764 26925547 10.1111/cge.12764

[14] Udagawa T, Jo T, Yanagihara T et al (2017) Altered expression of Crb2 in podocytes expands a variation of *CRB2* mutations in steroid-resistant nephrotic syndrome. Pediatr Nephrol 32:801–809. 10.1007/s00467-016-3549-4 27942854 10.1007/s00467-016-3549-4

[15] Sun Y, Kronenberg NM, Sethi SK et al (2025) CRB2 depletion induces YAP signaling and disrupts mechanosensing in podocytes. Am J Physiol Renal Physiol 328:F578–F595. 10.1152/ajprenal.00318.2024 40062402 10.1152/ajprenal.00318.2024 PMC12076484

