# Supplementary material for: Expanded CRB2-related disease phenotype: multisystem involvement and post-transplant complications in monozygotic twins
Source: Pediatr Nephrol. 2025 Jun 3;40(10):3093–9. doi: 10.1007/s00467-025-06827-w (PMC12401768; doi:10.1007/s00467-025-06827-w)
Supplement: Supplementary file 1 — Graphical abstract (PPTX 1.07 MB) [file 467_2025_6827_MOESM1_ESM.pptx]

## Slide 1
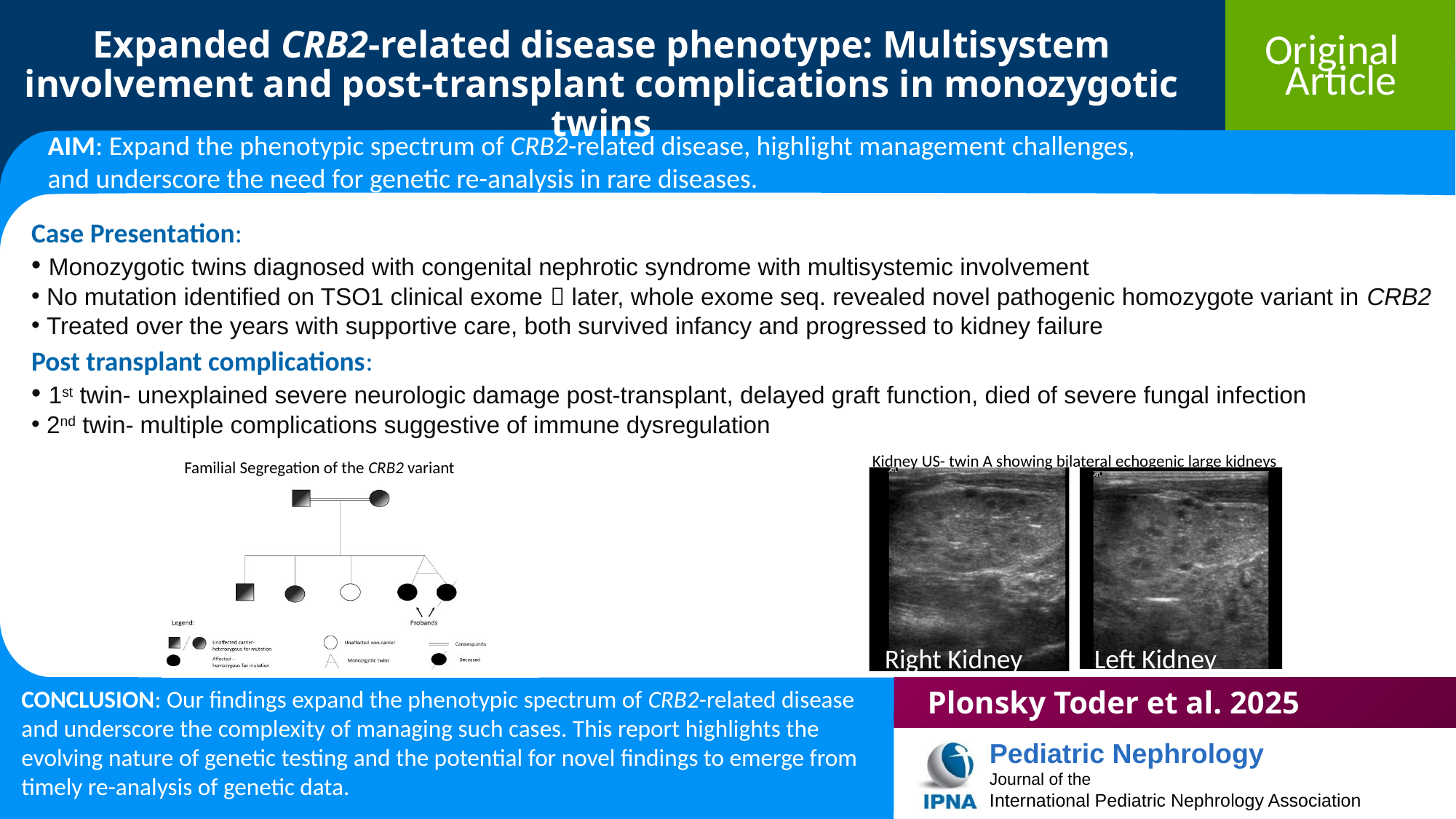

Expanded CRB2-related disease phenotype: Multisystem involvement and post-transplant complications in monozygotic twins
AIM: Expand the phenotypic spectrum of CRB2-related disease, highlight management challenges,
and underscore the need for genetic re-analysis in rare diseases.
Case Presentation:
 Monozygotic twins diagnosed with congenital nephrotic syndrome with multisystemic involvement
 No mutation identified on TSO1 clinical exome  later, whole exome seq. revealed novel pathogenic homozygote variant in CRB2
 Treated over the years with supportive care, both survived infancy and progressed to kidney failure
Post transplant complications:
 1st twin- unexplained severe neurologic damage post-transplant, delayed graft function, died of severe fungal infection
 2nd twin- multiple complications suggestive of immune dysregulation
Kidney US- twin A showing bilateral echogenic large kidneys
Familial Segregation of the CRB2 variant
Right Kidney
Left Kidney
CONCLUSION: Our findings expand the phenotypic spectrum of CRB2-related disease and underscore the complexity of managing such cases. This report highlights the evolving nature of genetic testing and the potential for novel findings to emerge from timely re-analysis of genetic data.
Plonsky Toder et al. 2025
